# Prevalence of maternal mental illness among children and adolescents in the UK between 2005 and 2017: a national retrospective cohort analysis

**DOI:** 10.1016/S2468-2667(19)30059-3

**Published:** 2019-05-30

**Authors:** Kathryn M Abel, Holly Hope, Eleanor Swift, Rosa Parisi, Darren M Ashcroft, Kyriaki Kosidou, Cemre Su Osam, Christina Dalman, Matthias Pierce

**Affiliations:** a Centre for Women's Mental Health, Faculty of Biology, Medicine and Health Sciences, University of Manchester, Manchester, UK; b Centre for Pharmacoepidemiology and Drug Safety, Manchester Academic Health Science Centre, Faculty of Biology, Medicine and Health Sciences, University of Manchester, Manchester, UK; c Greater Manchester Mental Health NHS Foundation Trust, Manchester, UK; d Center for Epidemiology and Community Medicine, Stockholm, Sweden; e Department of Public Health Sciences, Karolinska Institute, Stockholm, Sweden

## Abstract

**Background:**

Little information exists about the prevalence of children exposed to maternal mental illness. We aimed to estimate the prevalence of children and adolescents exposed to maternal mental illness in the UK between 2005 and 2017 using primary care data.

**Methods:**

In this national retrospective cohort study, we included children aged 0–16 years born between Jan 1, 1991, and Dec 31, 2015, who were linked to their mothers and registered on the primary care Clinical Practice Research Datalink (CPRD) between 2005 and 2017. We extracted data on diagnosis, symptoms, and therapy from the CRPD to define the following maternal mental illnesses: depression, anxiety, non-affective psychosis, affective psychosis, eating disorders, personality disorders, alcohol misuse disorder, and substance misuse disorder. We also extracted data on socioeconomic status from the Index of Multiple Deprivation 2010 and data on ethnicity from the Hospital Episode Statistics dataset. The main outcome was prevalence of maternal mental illness. Prevalence was calculated for each 2-year period of childhood (from age 0–<2 to 14–<16 years) using marginal predictions from a logistic regression model. We used survival analysis to estimate the incidence and cumulative risk of children experiencing maternal mental illness by age 16 years.

**Findings:**

We identified 783 710 children registered in the UK CPRD mother-baby link database, and included 547 747 children (381 685 mothers) in our analysis. Overall prevalence of maternal mental illness was 23·2% (95% CI 23·1–23·4), which increased during childhood (21·9%, 21·7–22·1 among the 0–<2 year age group *vs* 27·3%, 26·8–27·8 among the 14–<16 year age group). Depression and anxiety were the most prevalent maternal mental illnesses. The proportion of children exposed to maternal mental illness increased from 22·2% (21·9–22·4) between 2005 and 2007 to 25·1% (24·8–25·5) between 2015 and 2017. Geographically, the highest prevalence of maternal mental illness was observed in Northern Ireland (29·8%, 29·0–30·5). In England, prevalence of maternal mental illness was highest among children in the most deprived areas (28·3%, 27·8–28·8). The incidence of maternal mental illness was highest between 0–3 months (26·7 per 100 person years, 26·4–27·1). By age 16 years, the cumulative risk of maternal mental illness was 53·1% (52·8–53·3).

**Interpretation:**

One in four children aged 0–16 years are exposed to maternal mental illness and the prevalence of diagnosed and treated maternal mental illness is increasing. Policy makers and commissioners should consider this information and channel resources to target individuals in greatest need.

**Funding:**

The European Research Council and the National Institute for Health Research.

## Introduction

A 2010 EU report^1^ highlighted the importance of knowing more about children exposed to parental mental illness and the conditions in which they live. In 2011, the UK Government and the Social Care Institute for Excellence published a series of policy documents^2^ recognising that children exposed to parental mental illness are more likely to experience adversities compared with unexposed children, in addition to difficulties surrounding parental illness. Although premature mortality and long-term morbidity risks for offspring of individuals with mental illness can be high,^3^, ^4^, ^5^ more common and well recognised problems include adverse psychosocial development,^6^ and reversal of caregiving resulting in the developmental and psychological needs of children being neglected (so-called parentification^7^, ^8^).

Gopfert and colleagues^9^ previously reported that at least a quarter of adults admitted to acute psychiatric inpatient settings in England and Wales had dependent children. Other analyses^10^ have focused on the prevalence of mental illness in parents (mainly in the perinatal period). These statistics provide relevant information for service use planning and provide an insight into the association between parenthood and mental health. However, these previous analyses did not directly quantify the number of children exposed to parental mental illness. Without such information, policy makers and service planners cannot target resources effectively towards children with the most need.

**Research in context**

**Evidence before this study**

We systematically searched PsycINFO, Embase, MEDLINE, and PsychArticles for original research articles published between Jan 1, 1970, and April 2, 2019, reporting the prevalence of children exposed to parental mental illness using the search terms “prevalence” AND (“children” OR “offspring” OR “preschool” OR “infant” OR “baby” OR “adolescent” OR “teen) AND (“parent” OR “father” OR ”mother” OR “maternal” OR “paternal”) AND (“mental illness” OR “psychiatric disorder” OR “depression” OR “depressive” OR “mood disorders” OR “anxiety” OR “neurotic” OR “affective disorder” OR “schizophrenia” OR “bipolar” OR “psychosis” OR “psychotic” OR “substance abuse” OR “alcohol abuse” OR “alcohol misuse” OR “substance misuse” OR “eating disorder” OR “personality disorder”). The search identified 2097 non-duplicate articles. Most studies focused on the prevalence of mothers or fathers with mental illness, mostly in the perinatal period. Only two studies estimated the number of children and adolescents with parental mental illness: one survey of 37 000 Canadian households estimated the prevalence was 12%, and an Australian study of three separate data sources (two surveys and one health register) estimated the prevalence was 23%. Both studies relied on self-reported measures of mental illness, and thus might have been affected by responder bias. In the UK, no equivalent study exists, and it remains unclear whether the number of children exposed to parental mental illness is increasing and whether substantial geographical disparities in disease burden exist. This uncertainty makes it difficult for policy makers, commissioners, and service planners to design and implement policies to improve the lives of these children.

**Added value of this study**

To the best of our knowledge, this is the first study to use a large, nationally representative primary care database to calculate the prevalence of children aged 0–16 years with mothers with serious and common maternal mental illnesses between 2005 and 2017. We estimated that almost one in four children has a mother with an existing mental illness, the majority of whom have depression and anxiety, and by the time children reach 16 years, they have a 53% risk of maternal mental illness. The highest prevalence of maternal mental illness was observed among children aged 14–16 years between 2015 and 2017. This increase in maternal mental illness over time and with increasing age was observed for common mental disorders (ie, depression and anxiety) and serious mental illness (ie, affective psychotic disorder and non-affective psychotic disorder). We also investigated the geographical and demographic distribution of maternal mental illness. The prevalence of children with maternal mental illness was highest in regions with the most deprivation.

**Implications of all the available evidence**

These data highlight the need for long-term planning of high quality public health initiatives for children who experience maternal mental illness throughout childhood, not just in the perinatal period, and in regions with the highest disease burden. As the number of children with maternal mental illness continues to increase, health records and linkage to survey data should be used more extensively to provide more reliable information, which can guide policy and programmes to address the problems faced by these young people.

Accurate, up-to-date information about the numbers and ages of children and adolescents living with parental mental illness in the UK throughout childhood is scarce. In this study, we used data from the Clinical Practice Research Datalink (CPRD)^11^ to describe the prevalence of maternal mental illness, including common mental disorders, among children and adolescents (aged 0–16 years) in the UK.

## Methods

### Study design and participants

For this national retrospective cohort analysis, we extracted data from the CPRD database, which includes anonymised electronic health records from approximately 10% of general practices in the UK.^11^ The CPRD contains primary care data on clinical consultations, referrals to external health-care services, and therapies administered; therefore, our indicators describe treated prevalence. Entries made by general practitioners are meaningfully coded using the Read Code framework to enable the identification of medical events.^11^

Linkage of health data between parents and offspring is not done routinely in England. We used CPRD's mother-baby link, which matches children registered at the same general practice to mothers with a delivery date within 60 days of the child's birthday who share a practice-specific family identifier, as described previously.^12^

We derived a cohort of children included in the UK CPRD mother-baby link database born between Jan 1, 1991, and Dec 31, 2015. Follow-up was from birth or 2 years before birth until the earliest of: the child's 16th birthday, mother transferred out of practice, mother's death, end of data collection (Dec 31, 2017), or date the practice stopped collecting data. Each child was followed up for a minimum of 2 years.

Child-mother pairs were excluded if the mother was not registered at a practice for the first 2 years of the child's life, if mothers ended follow-up before Jan 1, 2007, if she was lost to follow-up, or if follow-up of the mother was missing for all 2 year age groups for her child.

To obtain accurate measures of socioeconomic status, eligible children were linked to the Index of Multiple Deprivation 2010 (IMD 2010).^13^ The IMD 2010 indicates deprivation at the area level and is linked using geographical data on the basis of a child's residential address. The IMD 2010 consists of 37 indicators for deprivation across seven domains. Areas are ranked and divided into quintiles from least to most deprived. Ethnicity data on children were collected from the Hospital Episode Statistics^14^ and the CPRD.^11^ The Hospital Episode Statistics dataset contains data provided during hospital visits and was previously validated using other linked data sources.^15^ Thus, when data on child ethnicity were available from both Hospital Episode Statistics and CPRD datasets, data from the Hospital Episode Statistics were prioritised. The IMD and Hospital Episode Statistics datasets only cover England and practices that consented to linkage (57% of practices).

This study was approved by the Independent Scientific Advisory Committee for CPRD research (17_187). The requirement for informed consent was waived because the study used de-identified patient data.

### Procedures

The key outcome measure was prevalence of maternal mental illness. Maternal mental illness was identified if there was evidence in the database of the following ICD-10 categories: non-affective psychosis (ICD 10 codes F20–4, F28–9); affective psychosis, including bipolar disorder (F25, F30–1); depressive disorders (F32–9); anxiety disorders, including obsessive-compulsive and post-traumatic stress disorders (F40–8); eating disorder (F50–3); personality disorders (F60–3); substance and alcohol dependence disorders (F10–19); and other psychiatric disorders not otherwise specified (F99). Acute alcohol intoxication (F10·0) and acute stress reaction (F43·0) were excluded.

Four data fields were used to classify mental disorder: diagnosis of a mental disorder during clinical consultation (including antecedent diseases, eg alcoholic cirrhosis of the liver); symptoms of mental disorder recorded during a consultation; referral to psychiatric care services; and prescription of psychotropic medications. The dates of these medical events were also obtained. Diagnostic and symptom codes were identified using previously published lists, or by searching the Read Code list using relevant strings and stubs (appendix p 12). Symptom codes were mapped to diagnostic categories. Medications were extracted using relevant British National Formulary chapters and each class of medication was assigned to their primary indication: antidepressants (depressive disorder); antipsychotics (non-affective psychosis), anxiolytics or hypnotics (anxiety disorders); mood stabilisers (affective psychosis); and drugs used to treat substance dependence. Code lists were finalised and assigned specific mental disorders using input from four clinical experts (ES, KK, DMA, and KMA) and uploaded to an online repository.

A primary care consultation with a diagnosis or recording of a mental health service contact was considered sufficient for indicating mental illness in mothers. However, general practitioners have been increasingly recording mental illness symptoms instead of diagnoses when a patient reports to general practice.^16^ Therefore, neither a symptom nor a medication alone was considered sufficient to indicate a mental illness: a symptom might be below the diagnostic threshold (eg, low mood does not necessarily imply depression) and most psychotropic medications have multiple indications (eg, amitriptyline is used for the treatment of both depression and neuropathic pain). For a symptom or prescription to define mental illness, individuals were required to have a previous diagnosis of the assigned mental illness or co-occurrence of a symptom and prescription within 3 months that pertained to the same mental illness. For these individuals, date of diagnosis was the earliest of the symptom and prescription date.

Maternal age at child's birth was categorised as younger than 20 years, 20–24 years, 25–29 years, 30–34 years, 35–39 years, and 40 years or older. Region was defined by the location of the general practice, divided into 11 regions of England, and the other UK countries: Scotland, Northern Ireland, and Wales.

### Statistical analysis

Period prevalence was defined as the proportion of children who had a mother with a maternal mental illness event occurring within a 2-year period, defined by the child's age group (0–<2, 2–<4, 4–<6, 6–<8, 8–<10, 10–<12, 12–<14, and 14–<16 years; the 2 year period before birth was used as the reference group). Children were included in age group calculations if their mother was followed up for the entirety of that 2-year age group and classified as exposed if an event indicating maternal mental illness occurred. The classification allowed for children to be included in more than one mental illness category for any age group (eg, anxiety and depression) and to change categories during follow-up. Because we included children born starting from Jan 1, 1991, period prevalence was calculated from Jan 1, 2005, to ensure children of all age groups were represented in each year—ie, in 2005, child ages ranged from 0 to 14 years.

The prevalence of maternal mental illness for each 2-year age group was estimated using marginal predictions from a logistic regression model, with age group as the exposure variable. The model accounted for multiple children belonging to one mother by allowing for clustering by maternal sibships in the calculation of standard errors. To account for uneven distribution of age groups between periods and secular changes in coding, we adjusted for the year when the age group began (categorical variable). Additionally, mothers transferring out of the reporting practice during follow-up might be a selective group, with higher mental illness risk. To reduce potential selection bias from censoring, stabilised inverse-probability weights were calculated that included the region, time enrolled with clinical practice, and quintile of deprivation, adapting the method of Howe and colleagues.^17^ Details of the model used to calculate stabilised inverse-probability weights, adjusted and unadjusted estimates, and prevalence by maternal mental illness and method of identification in CPRD are presented in the appendix. Prevalence estimates were recalculated for strata of the following covariates: maternal age at birth, region, and ethnicity.

Two further statistics were estimated in a time-to-event framework. For this analysis, the start of follow-up was defined from birth and we censored analysis at the earliest occurrence of end of follow-up (as previously defined) or a maternal mental illness event. We estimated the incidence of first maternal mental illness for age groups 0–<3, 3–<6, and 6–<12 months, and then yearly thereafter up to age 16 years. Incidence was defined as the rate of first maternal mental illness during each age band. We also used the Kaplan-Meier approach to calculate the cumulative risk of maternal mental illness by the time children had reached age 16 years. Analyses were done using Stata (version MP 15.1) and R (version 3.4.1).

### Role of the funding source

The study funders had no role in study design, data collection, data analysis, data interpretation, or writing of the report. MP, HH, DMA, and RP had access to the raw data. The corresponding author had full access to all the data and the final responsibility for the decision to submit for publication.

## Results

**Figure 1:**
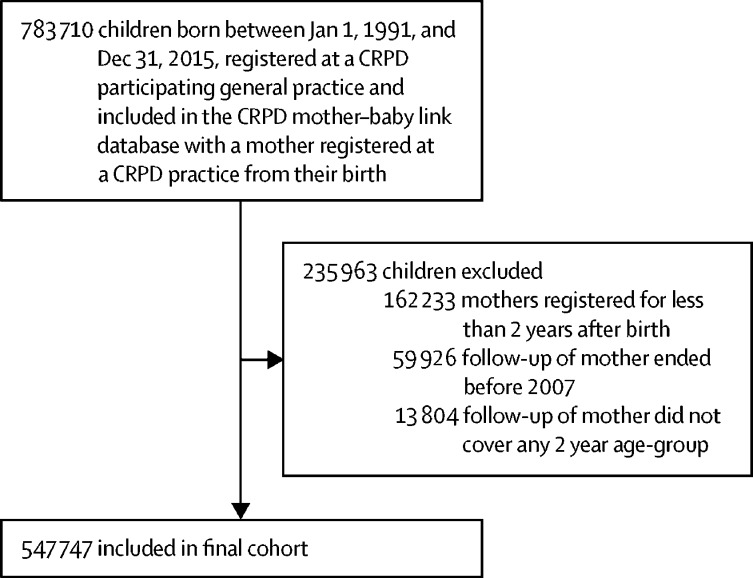
Study flow diagram CPRD= Clinical Practice Research Datalink.

783 710 children born between Jan 1, 1991, and Dec 31, 2015, were identified in the UK CPRD mother-baby link database. 235 963 children were excluded because their mother was registered at a general practice for less than 2 years after birth (n=162 233), their mother ended follow-up before Jan 1, 2007 (n=59 926), or follow-up of their mothers did not cover any 2 year age group (n=13 804). The final analysis cohort comprised 547 747 children and 381 685 mothers (figure 1). The median duration of follow-up was 7·9 years (IQR 4·5–12·6).

**Figure 2:**
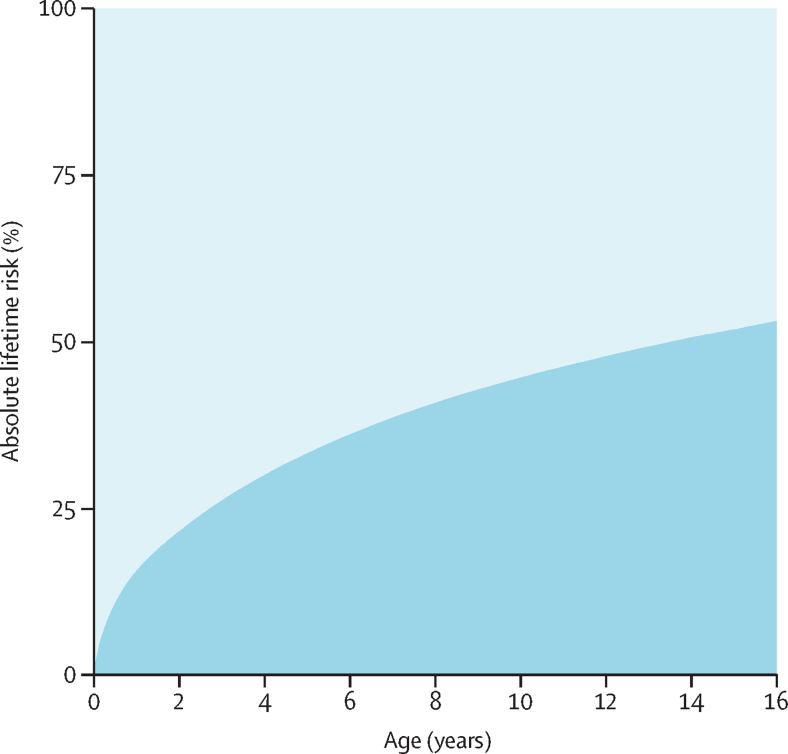
Absolute cumulative risk of maternal mental illness by age of child All 95% CIs were within 1 decimal place of estimates and thus have not been shown.

**Figure 3:**
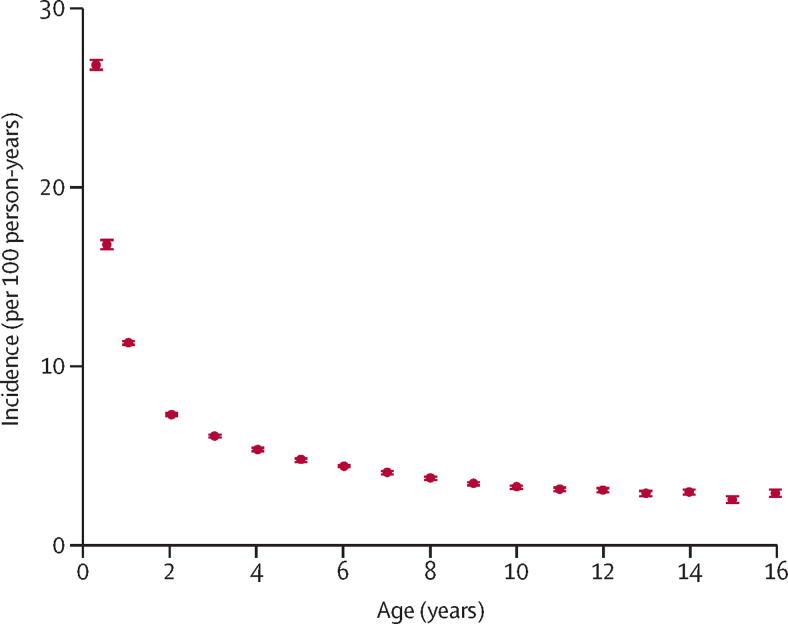
Estimated incidence of first observed maternal mental illness by age of child Incidence was estimated for age groups 0–<3, 3–<6, and 6–<12 months and then yearly thereafter up to age 16 years. Bars show 95% CIs.

Overall, 23·2% of children (95% CI 23·1–23·4) were exposed to maternal mental illness (table 1). The prevalence of maternal mental illness increased with increasing child age (21·9%, 21·7–22·1% for the 0–<2 year age group *vs* 27·3%, 26·8–27·8 for the 14–<16 year age group). The prevalence of maternal mental illness during all points in childhood was substantially higher than in the 2 years before birth (16·3%, 16·1–16·5). By age 16 years, 53·1% of children (52·8–53·3) had been exposed to maternal mental illness (figure 2). Incidence of first exposure to a maternal mental illness event was highest in the first 3 months of life (figure 3), with an incidence of 26·7 per 100 person-years (95% CI 26·4–27·1), which decreased to 3·8 per 100 person-years by age 10 years (3·7–3·9), and 2·6 per 100 person-years by age 16 years (2·4–2·7).

**Table 1:** Prevalence of maternal mental disorders by child age group

	**n**	**Any**	**Non-affective psychosis**	**Affective psychosis**	**Depression**	**Anxiety**	**Eating disorder**	**Personality disorder**	**Alcohol misuse**	**Substance misuse**
All ages	547 747	23·2% (23·1–23·4)	0·17% (0·15–0·18)	0·30% (0·28–0·32)	18·4% (18·3–18·6)	7·9% (7·9–8·0)	0·13% (0·12–0·14)	0·10% (0·09–0·11)	0·24% (0·23–0·26)	0·25% (0·23–0·26)
0–<2 years	345 983	21·9% (21·7–22·1)	0·13% (0·11–0·14)	0·22% (0·21–0·24)	17·6% (17·4–17·7)	6·5% (6·4–6·6)	0·14% (0·12–0·16)	0·11% (0·09–0·12)	0·12% (0·11–0·13)	0·22% (0·21–0·24)
2–<4 years	294 005	22·2% (22·0–22·3)	0·14% (0·12–0·15)	0·24% (0·22–0·26)	17·5% (17·3–17·6)	7·2% (7·1–7·3)	0·12% (0·10–0·14)	0·10% (0·08–0·12)	0·18% (0·16–0·19)	0·26% (0·23–0·28)
4–<6 years	249 898	22·8% (22·7–23·0)	0·16% (0·14–0·18)	0·29% (0·27–0·32)	18·0% (17·8–18·2)	7·9% (7·8–8·0)	0·13% (0·11–0·15)	0·09% (0·07–0·11)	0·22% (0·20–0·24)	0·26% (0·24–0·29)
6–<8 years	207 466	23·6% (23·4–23·8)	0·18% (0·16–0·20)	0·32% (0·29–0·35)	18·7% (18·5–18·9)	8·4% (8·3–8·6)	0·12% (0·09–0·14)	0·09% (0·07–0·11)	0·28% (0·26–0·31)	0·25% (0·23–0·28)
8–<10 years	166 024	24·2% (23·9–24·4)	0·20% (0·18–0·23)	0·37% (0·33–0·40)	19·3% (19·1–19·5)	8·8% (8·7–9·0)	0·11% (0·08–0·14)	0·09% (0·06–0·11)	0·34% (0·31–0·37)	0·26% (0·23–0·29)
10–<12 years	127 168	25·0% (24·7–25·2)	0·22% (0·18–0·25)	0·37% (0·33–0·42)	20·0% (19·8–20·3)	9·3% (9·2–9·5)	0·10% (0·07–0·13)	0·08% (0·05–0·11)	0·40% (0·36–0·45)	0·24% (0·20–0·27)
12–<14 years	95 102	25·9% (25·6–26·3)	0·24% (0·19–0·28)	0·41% (0·36–0·47)	20·9% (20·6–21·3)	10·0% (9·8–10·3)	0·13% (0·11–0·14)	0·10% (0·09–0·11)	0·45% (0·39–0·51)	0·26% (0·21–0·30)
14–<16 years	70 347	27·3% (26·8–27·8)	0·29% (0·22–0·36)	0·42% (0·35–0·50)	22·2% (21·7–22·6)	10·8% (10·4–11·1)	0·14% (0·13–0·16)	0·10% (0·09–0·12)	0·49% (0·42–0·57)	0·23% (0·17–0·29)
2 years before birth	196 372	16·3% (16·1–16·5)	0·08% (0·07–0·10)	0·14% (0·12–0·16)	11·4% (11·2–11·5)	5·5% (5·3–5·6)	0·15% (0·13–0·17)	0·08% (0·07–0·09)	0·13% (0·12–0·15)	0·26% (0·23–0·28)

Data are n, or estimated prevalence (95% CI). Prevalence was adjusted for calendar year.

**Figure 4:**
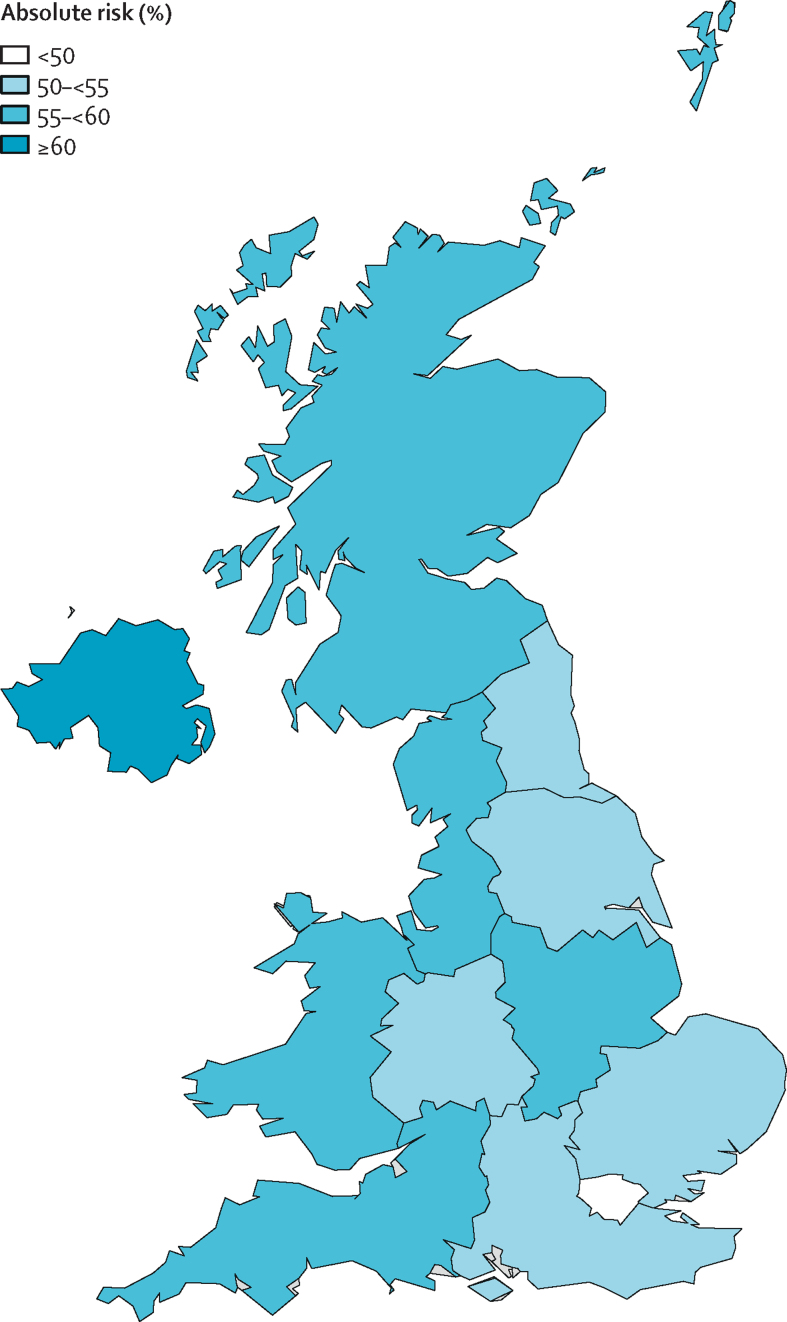
Absolute cumulative risk of exposure to maternal mental illness by age 16 years by geographical location

The prevalence of children exposed to maternal mental illness at any age varied by region; the proportion of children exposed to maternal mental illness was highest in Northern Ireland (29·8%), Scotland (26·0%), Wales (24·7%), the East Midlands (25·4%), the south west of England (25·3%), and the north west of England (25·1%; table 2). The proportion of children exposed to maternal mental illness was lowest in London (16·8%), the south east of England (21·2%), and eastern (20·9%) regions. The cumulative risk of children exposed to maternal mental illness by geographical region is shown in figure 4. Of the 320 762 children with linkage to England's IMD 2010, the prevalence of maternal mental illness ranged from 28·3% (95% CI 27·8–28·8) for children living in areas with the highest levels of deprivation to 18·0% (17·7–18·3) in areas with the lowest levels of deprivation.

**Table 2:** Prevalence of maternal mental illness by characteristics of mothers and children

	**n**	**Any**	**Non-affective psychosis**	**Affective psychosis**	**Depression**	**Anxiety**	**Eating disorder**	**Personality disorder**	**Alcohol misuse**	**Substance misuse**
Year*										
2005–07	158 794	22·2% (21·9–22·4)	0·16% (0·13–0·19)	0·25% (0·21–0·28)	17·7% (17·5–18·0)	7·3% (7·1–7·4)	0·15% (0·12–0·17)	0·06% (0·04–0·07)	0·22% (0·19–0·25)	0·24% (0·21–0·27)
2006–08	168 093	22·1% (21·8–22·4)	0·13% (0·11–0·16)	0·22% (0·19–0·25)	17·6% (17·4–17·8)	7·2% (7·0–7·3)	0·14% (0·12–0·16)	0·05% (0·04–0·07)	0·25% (0·22–0·28)	0·25% (0·22–0·28)
2007–09	175 970	22·3% (22·0–22·5)	0·15% (0·12–0·17)	0·24% (0·21–0·27)	17·7% (17·5–17·9)	7·4% (7·2–7·6)	0·17% (0·14–0·19)	0·05% (0·04–0·07)	0·26% (0·23–0·29)	0·26% (0·23–0·29)
2008–10	183 601	22·3% (22·1–22·6)	0·14% (0·11–0·16)	0·26% (0·23–0·29)	17·7% (17·4–17·9)	7·4% (7·2–7·5)	0·15% (0·13–0·17)	0·07% (0·06–0·09)	0·28% (0·25–0·31)	0·23% (0·20–0·26)
2009–11	184 409	23·0% (22·7–23·2)	0·17% (0·14–0·19)	0·27% (0·24–0·30)	18·3% (18·1–18·6)	7·6% (7·5–7·8)	0·15% (0·13–0·18)	0·07% (0·05–0·08)	0·24% (0·21–0·26)	0·24% (0·21–0·27)
2010–12	188 881	22·9% (22·6–23·1)	0·15% (0·13–0·18)	0·29% (0·26–0·32)	18·2% (17·9–18·4)	7·6% (7·4–7·7)	0·13% (0·11–0·15)	0·09% (0·07–0·11)	0·27% (0·24–0·30)	0·23% (0·20–0·26)
2011–13	181 249	23·4% (23·2–23·7)	0·17% (0·15–0·20)	0·32% (0·28–0·35)	18·6% (18·3–18·8)	8·0% (7·9–8·2)	0·11% (0·09–0·13)	0·09% (0·07–0·11)	0·25% (0·22–0·28)	0·26% (0·23–0·29)
2012–14	168 552	23·2% (22·9–23·4)	0·18% (0·15–0·21)	0·32% (0·29–0·36)	18·4% (18·2–18·6)	8·1% (7·9–8·2)	0·11% (0·09–0·13)	0·09% (0·07–0·11)	0·24% (0·21–0·27)	0·27% (0·24–0·30)
2013–15	145 574	24·2% (24·0–24·5)	0·19% (0·16–0·22)	0·36% (0·32–0·39)	19·3% (19·1–19·6)	8·5% (8·3–8·7)	0·11% (0·09–0·13)	0·13% (0·11–0·16)	0·22% (0·19–0·25)	0·27% (0·24–0·31)
2014–16	108 298	24·4% (24·0–24·7)	0·20% (0·17–0·24)	0·32% (0·27–0·36)	19·5% (19·2–19·8)	8·8% (8·6–9·0)	0·11% (0·09–0·14)	0·17% (0·14–0·20)	0·23% (0·20–0·27)	0·23% (0·19–0·26)
2015–17	88 944	25·1% (24·8–25·5)	0·20% (0·16–0·24)	0·37% (0·32–0·42)	20·1% (19·8–20·4)	9·4% (9·2–9·6)	0·11% (0·08–0·14)	0·21% (0·16–0·25)	0·22% (0·19–0·26)	0·23% (0·19–0·27)
**Index of Multiple Deprivation quintile**†										
1	77 353	18·0% (17·7–18·3)	0·13% (0·09–0·17)	0·24% (0·20–0·29)	13·6% (13·3–13·9)	6·1% (6·0–6·3)	0·11% (0·09–0·13)	0·06% (0·05–0·08)	0·14% (0·12–0·17)	0·08% (0·06–0·11)
2	67 503	20·2% (19·9–20·6)	0·13% (0·09–0·17)	0·24% (0·19–0·30)	15·8% (15·4–16·1)	6·7% (6·5–6·9)	0·11% (0·08–0·13)	0·08% (0·06–0·10)	0·19% (0·16–0·22)	0·10% (0·07–0·13)
3	60 827	23·0% (22·5–23·4)	0·12% (0·09–0·15)	0·24% (0·19–0·28)	18·2% (17·8–18·6)	7·7% (7·4–7·9)	0·14% (0·12–0·17)	0·09% (0·07–0·11)	0·21% (0·17–0·24)	0·18% (0·14–0·22)
4	60 438	25·3% (24·9–25·8)	0·17% (0·13–0·21)	0·35% (0·29–0·41)	20·6% (20·2–21·0)	8·1% (7·8–8·3)	0·17% (0·13–0·20)	0·16% (0·12–0·19)	0·28% (0·24–0·33)	0·26% (0·22–0·31)
5	54 641	28·3% (27·8–28·8)	0·26% (0·20–0·32)	0·40% (0·33–0·47)	23·0% (22·6–23·5)	9·1% (8·9–9·4)	0·15% (0·11–0·18)	0·15% (0·11–0·18)	0·38% (0·33–0·43)	0·56% (0·48–0·64)
**Ethnicity of child**‡										
White	340 462	24·6% (24·4–24·8)	0·15% (0·14–0·17)	0·31% (0·28–0·33)	19·7% (19·5–19·8)	8·3% (8·2–8·4)	0·14% (0·13–0·15)	0·11% (0·10–0·12)	0·24% (0·23–0·26)	0·26% (0·24–0·28)
Asian or British Asian	19 389	10·2% (9·7–10·7)	0·30% (0·19–0·41)	0·25% (0·15–0·34)	7·3% (6·8–7·8)	3·1% (2·8–3·3)	0·02% (0·01–0·03)	0·01% (0·00–0·02)	0·04% (0·01–0·06)	0·06% (0·02–0·09)
Mixed	10 755	21·4% (20·5–22·3)	0·24% (0·11–0·37)	0·28% (0·18–0·39)	16·8% (16·0–17·6)	6·9% (6·4–7·4)	0·16% (0·10–0·23)	0·15% (0·08–0·23)	0·29% (0·20–0·39)	0·22% (0·14–0·31)
Black or black British	9025	10·3% (9·6–11·0)	0·33% (0·18–0·47)	0·29% (0·13–0·45)	7·1% (6·5–7·7)	2·7% (2·4–3·1)	0·03% (0·00–0·06)	0·06% (0·02–0·10)	0·12% (0·05–0·19)	0·09% (0·03–0·14)
Other	5398	14·4% (13·4–15·4)	0·20% (0·08–0·32)	0·33% (0·13–0·52)	10·7% (9·9–11·6)	4·8% (4·2–5·4)	0·13% (0·06–0·19)	0·07% (0·01–0·14)	0·08% (0·02–0·14)	0·19% (0·07–0·32)
**Age of mother at birth (years)**										
<20	19 064	31·9% (31·3–32·6)	0·19% (0·13–0·26)	0·35% (0·26–0·45)	25·8% (25·2–26·4)	10·1% (9·7–10·5)	0·29% (0·23–0·35)	0·34% (0·26–0·41)	0·28% (0·22–0·34)	0·61% (0·50–0·72)
20–24	77 740	29·7% (29·4–30·1)	0·18% (0·14–0·21)	0·37% (0·32–0·41)	24·1% (23·8–24·4)	10·1% (9·9–10·3)	0·24% (0·21–0·27)	0·20% (0·17–0·23)	0·28% (0·25–0·32)	0·47% (0·41–0·53)
25–29	140 776	24·5% (24·3–24·8)	0·15% (0·13–0·18)	0·31% (0·27–0·34)	19·6% (19·3–19·8)	8·7% (8·5–8·8)	0·15% (0·13–0·16)	0·11% (0·09–0·12)	0·25% (0·23–0·28)	0·30% (0·27–0·33)
30–34	177 946	20·5% (20·3–20·7)	0·16% (0·14–0·18)	0·25% (0·22–0·27)	16·2% (16·0–16·4)	7·1% (7·0–7·2)	0·10% (0·09–0·11)	0·07% (0·06–0·08)	0·22% (0·20–0·24)	0·16% (0·15–0·18)
35–39	106 553	20·3% (20·0–20·6)	0·18% (0·14–0·21)	0·30% (0·26–0·34)	15·9% (15·7–16·2)	6·7% (6·6–6·9)	0·07% (0·05–0·08)	0·04% (0·03–0·05)	0·22% (0·20–0·24)	0·12% (0·11–0·14)
40–49	25 668	21·2% (20·7–21·7)	0·24% (0·16–0·31)	0·32% (0·25–0·40)	16·9% (16·4–17·4)	6·9% (6·6–7·2)	0·04% (0·02–0·05)	0·05% (0·03–0·07)	0·28% (0·22–0·33)	0·12% (0·08–0·15)
**Region of the UK**										
North West	66 494	25·1% (24·7–25·5)	0·20% (0·16–0·25)	0·34% (0·28–0·40)	19·5% (19·1–19·9)	9·5% (9·2–9·7)	0·13% (0·10–0·16)	0·11% (0·08–0·13)	0·36% (0·32–0·41)	0·33% (0·28–0·38)
South Central	64 885	22·7% (22·2–23·1)	0·15% (0·11–0·19)	0·30% (0·25–0·35)	18·2% (17·8–18·6)	7·3% (7·0–7·5)	0·15% (0·12–0·18)	0·11% (0·08–0·13)	0·20% (0·16–0·24)	0·23% (0·18–0·28)
South East coast	54 820	21·2% (20·8–21·6)	0·13% (0·09–0·17)	0·29% (0·23–0·36)	17·0% (16·6–17·4)	6·5% (6·3–6·7)	0·10% (0·08–0·13)	0·11% (0·08–0·14)	0·19% (0·16–0·23)	0·11% (0·08–0·14)
London	50 820	16·8% (16·4–17·2)	0·16% (0·12–0·21)	0·26% (0·21–0·32)	12·3% (11·9–12·7)	5·6% (5·3–5·8)	0·10% (0·07–0·12)	0·07% (0·05–0·09)	0·18% (0·14–0·22)	0·08% (0·05–0·10)
West Midlands	49 990	22·9% (22·5–23·4)	0·09% (0·06–0·12)	0·23% (0·18–0·28)	18·1% (17·7–18·6)	7·5% (7·3–7·8)	0·11% (0·09–0·14)	0·10% (0·07–0·13)	0·22% (0·17–0·26)	0·25% (0·20–0·30)
Wales	50 260	24·7% (24·3–25·2)	0·23% (0·17–0·28)	0·28% (0·22–0·33)	20·0% (19·6–20·4)	8·5% (8·2–8·7)	0·13% (0·10–0·15)	0·07% (0·05–0·09)	0·25% (0·20–0·30)	0·30% (0·25–0·35)
Scotland	48 954	26·0% (25·5–26·5)	0·16% (0·12–0·20)	0·38% (0·32–0·44)	21·5% (21·0–22·0)	9·3% (9·0–9·6)	0·14% (0·11–0·17)	0·12% (0·10–0·14)	0·38% (0·32–0·44)	0·56% (0·48–0·64)
East of England	47 522	20·9% (20·4–21·4)	0·20% (0·13–0·27)	0·29% (0·22–0·36)	16·2% (15·7–16·6)	6·7% (6·5–7·0)	0·16% (0·13–0·19)	0·13% (0·09–0·17)	0·16% (0·12–0·20)	0·12% (0·09–0·15)
South West	47 522	25·3% (24·8–25·8)	0·15% (0·11–0·20)	0·28% (0·22–0·34)	20·9% (20·4–21·4)	7·7% (7·4–8·0)	0·15% (0·11–0·18)	0·09% (0·06–0·11)	0·20% (0·16–0·24)	0·24% (0·19–0·29)
Northern Ireland	20 320	29·8% (29·0–30·5)	0·21% (0·12–0·30)	0·30% (0·22–0·38)	24·0% (23·3–24·8)	13·0% (12·5–13·5)	0·12% (0·08–0·17)	0·07% (0·04–0·10)	0·31% (0·24–0·38)	0·18% (0·13–0·24)
East Midlands	19 418	25·4% (24·5–26·2)	0·21% (0·08–0·33)	0·37% (0·25–0·49)	20·0% (19·2–20·8)	10·8% (10·2–11·4)	0·16% (0·10–0·23)	0·16% (0·06–0·27)	0·25% (0·13–0·37)	0·21% (0·10–0·33)
Yorkshire and the Humber	16 270	22·2% (21·3–23·2)	0·18% (0·07–0·29)	0·15% (0·07–0·23)	17·6% (16·7–18·5)	6·6% (6·1–7·0)	0·10% (0·04–0·17)	0·05% (0·02–0·08)	0·16% (0·07–0·25)	0·19% (0·12–0·27)
North East	10 350	24·8% (23·7–25·8)	0·19% (0·08–0·30)	0·33% (0·20–0·46)	19·1% (18·1–20·1)	9·6% (8·9–10·3)	0·13% (0·06–0·19)	0·09% (0·03–0·14)	0·25% (0·17–0·34)	0·49% (0·29–0·69)

Data are n, or estimated prevalence (95% CI).

* Some children were included in more than one time period.

† Quintile 1 corresponds to areas with the least deprivation and quintile 5 corresponds to areas with the highest level of deprivation. Data missing for 226 985 (41%) of 547 747 children because their practice did not consent to linkage. Data was available for England only.

‡ Data missing for 162 718 (30%) of 547 747 children, mostly due to general practices not consenting to linkage to hospital records (13% of data missing among children with linkage).

The prevalence of maternal mental illness was higher in children born to mothers aged less than 20 years (31·9%, 95% CI 31·3–32·6) and 20–24 years (29·7%, 29·4–30·1) than children born to mothers aged 30–34 years (20·5%, 20·3–20·7) and 35–39 years (20·3%, 20·0–20·6). Of the 385 029 children with ethnicity data, 10·2% of Asian children, 10·3% of black children, and 24·6% of white children had maternal mental illness.

Depression and anxiety were the most prevalent maternal mental illnesses and were highest among children aged 14–<16 years (22·2%, 95% CI 21·7–22·6 for depression; 10·8%, 10·4–11·1 for anxiety; table 1). The prevalence of alcohol misuse (0·49%, 0·42–0·57), affective psychosis (0·42%, 0·35–0·50), and non-affective psychosis (0·29%, 0·22–0·36) was also highest among the mothers of children aged 14–<16 years. With the exception of substance misuse and eating disorders, the prevalence of all maternal mental illnesses was higher during childhood than in the 2 years before birth. With the exception of personality disorders, eating disorders, and substance misuse disorders, the prevalence of maternal mental illnesses increased with child age. For example, the proportion of children exposed to maternal non-affective psychosis increased from 0·13% (95% CI 0·11–0·14) for the 0–<2 year age group to 0·29% (0·22–0·36) for the 14–<16 year age group, and the proportion of children exposed to maternal anxiety increased from 6·5% (6·4–6·6) for the 0–<2 year age group to 10·8% (10·4–11·1) for the 14–<16 year age group.

The prevalence of children exposed to maternal mental illness increased over time from 22·2% (95% CI 21·9–22·4) between 2005 and 2007 to 25·1% (24·8–25·5) between 2015 and 2017 (table 2). Increases in prevalence were particularly marked for maternal depression, personality disorders, and affective psychosis, which included bipolar disorder. However, the increase in prevalence of non-affective psychosis was less marked.

## Discussion

This is, to our knowledge, the largest population-based study of the prevalence of UK children and adolescents exposed to maternal mental illness to date. The large sample size enabled calculation of reliable estimates of the number and ages of children exposed to maternal mental illness and the estimation of exposure to maternal mental illness throughout childhood, including the types of illness and regional differences. This is also the first study to investigate changes in prevalence by maternal diagnosis for the period 2005–17.

We report several key findings. First, we found that a high proportion of children are exposed to maternal mental illness. Prevalence of maternal mental illness was high despite women with mental illness being consistently found to have lower fertility than the general population.^18^ At present, one in four children in the UK aged 0–16 years are exposed to maternal mental illness. Our study supplements existing evidence—which describes an excess of incident cases of maternal mental illness during the first postnatal year^10^—and we report that risk of incident maternal mental illness persists across a child's life into their teenage years. By the time a child reaches age 16 years in the UK, there is a 53% chance that their mother has had a mental illness severe enough for a diagnosis and receipt of psychological or pharmaceutical therapies within primary care.

Second, we found that the number of children exposed to maternal mental illness, as shown by mothers seen in UK primary care, has increased substantially between 2005 and 2017. Between 2005 and 2017, the number of children of mothers with depression or anxiety increased and, although serious mental illness remains relatively rare, the number of children exposed to mothers treated for affective psychosis increased substantially, as did the numbers of children exposed to mothers with personality disorders. However, no increases in the number of children exposed to maternal eating disorders, or substance or alcohol dependence disorders were identified for the same time period.

Third, geography seems to affect the risk of childhood exposure to maternal mental illness. In particular, children had an absolute risk of 60% and over 55% of maternal mental illness by age 16 years, in Northern Ireland and the north west of England, respectively. By contrast, children born in London and the south east of England were least likely to experience maternal mental illness. The areas of the UK with the highest prevalence of maternal mental illness coincide with areas of the UK with the highest levels of deprivation and adult mental illness in general, as shown by previous research.^19^

Children born into poverty, or who are offspring of teenage mothers, were most likely to be exposed to maternal mental illness. By contrast, Asian and black children were less often exposed to maternal mental illness than were white children. For less severe disorders, utilisation of mental health services is lower for minority and migrant groups than for non-minority and non-migrant groups,^20^ which might be reflected in this treated prevalence sample. However, these groups are known to have a higher prevalence of serious mental illness, which was consistent with our data: the prevalence of treated maternal non-affective psychosis was two times higher in black (0·33%) and Asian children (0·30%) than white children (0·15%).

Our 2-year treated prevalence estimate (23·2%) is comparable to the estimated 1-year prevalence reported in an Australian study (23·3%).^21^ Both estimates are considerably higher than the 1-year weighted prevalence reported in a 2002 Canadian population survey (12·1%).^22^

There are several possible explanations for the observed increase in the number of children with mothers diagnosed and treated for mental illness within primary care over the study period. The increase might imply that more mothers are developing illnesses or it might mean that the same number of mothers might be becoming ill over time, but the hidden proportion (ie, mothers missed by primary care because they did not seek help or because they were not diagnosed), might be decreasing. However, the increased prevalence of common psychiatric disorders was also observed for severe mental illnesses, which are less likely to be hidden. Alternatively, thresholds for diagnoses might have changed over time. The increased exposure to treated maternal affective psychosis might reflect the use of lowered thresholds by general practitioners for the diagnosis of bipolar disorder. Analysis of Swedish data^23^ showed that the number of women contacting services with diagnosed bipolar disorder increased by four times between 1991 and 2010. Secular changes in CPRD coding could also explain observed increases over time. For example, the introduction of the Quality Outcomes Framework in 2004 incentivised general practitioners to monitor chronic illnesses, including mental illnesses.^24^ Severe mental illness was one of the outcomes included in the Quality Outcomes Framework and consultations for severe mental illness increased immediately after introduction of the Quality Outcomes Framework, and this effect did not stabilise until 2008.^25^ Therefore, the period prevalence for severe mental illness between 2005 and 2007 might be inflated.

The CPRD primary care registry database is one of the largest and most comprehensive electronic health registries worldwide. Over 99% of the UK population register with a general practitioner, who are notified of all diagnoses and therapies made in secondary care. Using routinely collected primary care data minimises selection, attrition, and information biases that can arise in observational studies, however, the CPRD database did have some limitations.

The most important limitation of the current CPRD dataset is the lack of reliable information about fathers since linkage to paternity has not yet been established. We believe this information is a public health imperative: emerging evidence indicates that paternal mental health might have important effects on children.^26^ A previous analysis^27^ estimated the incidence of paternal depression using UK primary care data by linking children to an adult male in the same household. In the future, similar linkages might be possible using the CPRD dataset. A further limitation is that the CPRD covers approximately 10% of the UK. Although the dataset is considered representative of the UK population in terms of age and ethnicity,^11^ some geographical areas are under-represented. Patients who transferred between CPRD reporting practices might have received a new identifier, and this would not be identified in our anonymous data extract. Therefore, it is possible that a small number of sibship linkages could have been missed and thus our SEs might be slightly underestimated.

To the best of our knowledge, this is the first study to use the CPRD mother-baby link to estimate prevalence of childhood exposure to maternal mental illness.^28^ We are confident that we have not overestimated the number of women with incident mental illness, because we used conservative assumptions to estimate child exposure. The accuracy of primary care records to identify mental illness was demonstrated by John and colleagues^29^ who compared Welsh primary care records with the Mental Health Inventory-5, a validated and reliable self-report measure of mental health. The authors tested a number of algorithms that combined available information in the general practitioner record and reported that the optimum algorithm to detect cases of mental illness triangulated diagnoses, symptoms, and therapy information and was highly specific (96%), with lower sensitivity (75%). Our study triangulated data similarly, suggesting that the high level of diagnosed and treated maternal mental illness observed might have underestimated the true burden.

Furthermore, our ability to cross-reference estimates with Swedish data (Pierce M, unpublished) on diagnosed mental disorders registered within high validity administrative health-care registries covering the total population provides us with the opportunity to validate our findings. Preliminary analyses by the authors in the Swedish health-care register show similar increases in treated prevalence of mainly common mental illnesses and bipolar disorder between 2006 and 2016 (Pierce M, unpublished).

Considering the importance of increasing prevalence of children living with maternal mental illness for policy makers and public mental health, replication of this study using other high quality datasets is needed to establish if the number of children exposed to maternal psychotic disorders constitutes a real population rise. However, a higher proportion of children are exposed to common, less severe mental illness; and our findings might suggest that more ill mothers are receiving treatment, which should improve circumstances for their children. Many women might have a dual diagnosis of depression and anxiety and diagnoses can change over time. Providing mothers with the correct diagnosis and treatments is of key importance. Children's needs are clearly distinct from their parents.^30^ Underlying environmental factors such as poverty or violence in the home are important sources of adversity for children exposed to maternal mental illness.^31^ Similarly, some common consequences of exposure to maternal mental illness, such as social and educational difficulties and reduced quality of life,^32^ are unlikely to be met by the limited services available for these young people. Adult, child, and adolescent mental health services are only available when children exposed to maternal mental illness become ill themselves. Furthermore, the absolute risk of developing severe mental illness, even when both parents have a diagnosis of severe mental illness, is low;^33^ therefore, solely increasing mental health provision is unlikely to meet the needs of these children over time.

Children exposed to maternal mental illness are an easily identifiable group of at-risk young people. Reliable, detailed information about the numbers, ages, regional variation, and types of illness provides vital information for researchers, policy makers, clinical commissioners, and education and health service providers. Appropriate and timely diversion of funds to areas of greatest need is now required. Such an approach fits well with UK Department of Health initiatives to make funding of health care and health research more representative of disease burden across the country^34^ and more focused on prevention.^35^ These concerns should not be confined to the UK; this population is increasingly recognised as in need of greater attention and better support across Europe and studies in the USA and Australia have also demonstrated that many children are exposed to maternal mental illness. Therefore, children exposed to maternal mental illness represent a challenge for the Global Health community. The lack of recognition of need in these children,^36^ and growth in the number of mothers diagnosed and treated for mental illness over time suggest that children exposed to maternal mental illness represent a substantial population of children and adolescents with unmet needs. Planned linkages to the hospital records of children exposed to maternal mental illness will better quantify these needs and provide valuable evidence about how best to address them.

Interventions and support must consider the particular needs of these parents and children. Additional investigation of preventive interventions that can be most helpful is warranted to assure appropriate resources are available to improve child outcomes. On the basis of currently available information, such investigations should consider children's concerns about stigma while recognising their specific needs to include interventions in addition to effective mental health treatment for their parents.
