# Supplementary material for: Prevalence of maternal mental illness among children and adolescents in the UK between 2005 and 2017: a national retrospective cohort analysis
Source: Lancet Public Health. 2019 May 30;4(6):e291–300. doi: 10.1016/S2468-2667(19)30059-3 (PMC6557735; doi:10.1016/S2468-2667(19)30059-3)
Supplement: Supplementary appendix [file mmc1.pdf]

THE LANCET
Public Health

Supplementary appendix

This appendix formed part of the original submission and has been peer reviewed. We post it as supplied by the authors.

Supplement to: Abel KM, Hope H, Swift E, et al. Prevalence of maternal mental illness among children and adolescents in the UK between 2005 and 2017: a national retrospective cohort analysis. *Lancet Public Health* 2019; **4:** e291–300.

Appendices

Appendix 1: Supplementary Tables of period prevalence by maternal mental illness, method of identification in CPRD, age and calendar year

Appendix 2: Data quality

Appendix 3: Resources and searches used to identify Read codes and Prod codes for case definition

Appendix 4 : Methods and models used for inverse-probability of missingness weights

Appendix 1: Supplementary Table 1 period prevalence by maternal mental illness, method of identification in CPRD, age and calendar year

1. **Any mental disorder**

	Diagnosis		Service use		Current Tx & Sx		Current Tx & historical Dx		Current Sx & historical Dx		Any	
**Age-range**	**N**	**%**	**N**	**%**	**N**	**%**	**N**	**%**	**N**	**%**	**N**	**%**
-2 to 0	19,610	*10.0*	12,615	*6.4*	9,529	*4.9*	19,679	*10.0*	8,817	*4.2*	32,208	*16.3*
0 to 2	50,871	*14.7*	28,933	*8.4*	27,164	*7.9*	50,426	*14.6*	21,453	*6.2*	74,607	*21.6*
2 to 4	35,476	*12.1*	23,782	*8.1*	21,314	*7.3*	45,625	*15.5*	17,664	*6.0*	63,889	*21.7*
4 to 6	29,241	*11.7*	19,928	*8.0*	17,729	*7.1*	41,385	*16.6*	15,383	*6.2*	55,839	*22.3*
6 to 8	23,459	*11.3*	16,469	*7.9*	14,463	*7.0*	36,312	*17.5*	12,864	*6.3*	47,620	*22.7*
8 to 10	18,164	*10.9*	13,047	*7.9*	11,329	*6.8*	30,409	*18.3*	10,376	*6.3*	38,782	*23.0*
10 to 12	13,678	*10.8*	9,779	*7.7*	8,564	*6.7*	24,172	*19.0*	7,871	*6.2*	30,340	*23.4*
12 to 14	10,100	*10.6*	7,205	*7.6*	6,429	*6.8*	18,583	*19.5*	6,019	*6.3*	22,981	*23.9*
14 to 16	7,451	*10.6*	5,192	*7.4*	4,785	*6.8*	14,126	*20.1*	4,592	*6.5*	17,283	*24.2*
**Year**												
2005-07	21,331	*13.4*	8375	*6.5*	8290	*6.5*	23,348	*14.7*	8,801	*5.5*	32,833	*20.5*
2006-08	21,535	*12.8*	9355	*6.7*	9198	*6.5*	24,930	*14.8*	9,688	*5.8*	34,833	*20.7*
2007-09	21,513	*12.2*	10016	*6.8*	9865	*6.6*	26,421	*15.0*	10,200	*5.8*	36,669	*20.9*
2008-10	22,094	*12.0*	11276	*7.3*	10441	*6.7*	27,833	*15.2*	10,770	*5.9*	38,453	*21.0*
2009-11	21,981	*11.9*	11958	*7.6*	11159	*7.1*	29,116	*15.8*	11,016	*6.0*	39,840	*21.5*
2010-12	22,106	*11.7*	12210	*7.5*	11496	*7.1*	30,241	*16.0*	11,426	*6.1*	41,126	*21.9*
2011-13	20,657	*11.4*	11811	*7.6*	11155	*7.2*	29,809	*16.5*	11,099	*6.1*	40,407	*22.3*
2012-14	18,889	*11.1*	12425	*8.5*	10399	*7.0*	27,760	*16.5*	10,109	*6.0*	37,445	*22.4*
2013-15	16,243	*11.2*	12181	*9.7*	9079	*7.2*	25,118	*17.3*	8,874	*6.1*	33,840	*23.4*
2014-16	12,000	*11.1*	9993	*10.0*	7375	*7.3*	19,510	*18.0*	6,780	*6.3*	25,904	*23.7*
2015-17	9,921	*11.2*	7455	*10.0*	5583	*7.3*	16,631	*18.7*	5,646	*6.4*	21,972	*25.2*

Tx= treatment; Sx = symptoms; Dx = diagnosis

2. Non-affective psychosis

	Diagnosis		Service use*		Current Tx & Sx		Current Tx & historical Dx		Current Sx & historical Dx		Any	
**Age-range**	**N**	**%**	**N**	**%**	**N**	**%**	**N**	**%**	**N**	**%**	**N**	**%**
-2 to 0	53	*0.03*	-	-	15	*0.01*	127	*0.06*	3	*0.00*	161	*0.08*
0 to 2	204	*0.06*	-	-	44	*0.01*	318	*0.09*	15	*0.00*	419	*0.12*
2 to 4	131	*0.04*	-	-	44	*0.01*	314	*0.11*	17	*0.01*	376	*0.13*
4 to 6	144	*0.06*	-	-	37	*0.01*	303	*0.12*	15	*0.00*	365	*0.15*
6 to 8	114	*0.05*	-	-	24	*0.01*	286	*0.14*	10	*0.00*	334	*0.16*
8 to 10	95	*0.06*	-	-	27	*0.02*	246	*0.15*	9	*0.01*	300	*0.18*
10 to 12	73	*0.06*	-	-	18	*0.01*	205	*0.16*	5	*0.00*	240	*0.19*
12 to 14	61	*0.06*	-	-	14	*0.01*	154	*0.16*	5	*0.01*	178	*0.19*
14 to 16	33	*0.05*	-	-	9	*0.01*	128	*0.18*	1	*0.00*	140	*0.20*
**Year**												
2005-07	78	*0.05*	-	-	23	*0.01*	193	*0.12*	10	*0.01*	225	*0.14*
2006-08	74	*0.04*	-	-	22	*0.01*	169	*0.10*	8	*0.00*	204	*0.12*
2007-09	70	*0.04*	-	-	22	*0.01*	193	*0.11*	12	*0.01*	222	*0.13*
2008-10	67	*0.04*	-	-	22	*0.01*	190	*0.10*	12	*0.01*	217	*0.12*
2009-11	85	*0.05*	-	-	26	*0.01*	225	*0.12*	9	*0.00*	263	*0.14*
2010-12	83	*0.04*	-	-	19	*0.01*	203	*0.11*	3	*0.00*	250	*0.13*
2011-13	103	*0.06*	-	-	19	*0.01*	221	*0.12*	8	*0.00*	273	*0.15*
2012-14	120	*0.07*	-	-	27	*0.02*	195	*0.12*	5	*0.00*	258	*0.15*
2013-15	104	*0.07*	-	-	22	*0.02*	196	*0.13*	6	*0.00*	246	*0.17*
2014-16	71	*0.06*	-	-	17	*0.02*	152	*0.14*	2	*0.00*	192	*0.18*
2015-17	53	*0.05*	-	-	13	*0.01*	144	*0.16*	5	*0.01*	163	*0.18*

Tx= treatment; Sx = symptoms; Dx = diagnosis * No codes were identified under this criterion for this mental illness.

3. **Affective psychosis**

	Diagnosis		Service use*		Current Tx & Sx		Current Tx & historical Dx		Current Sx & historical Dx		Any	
**Age-range**	**N**	**%**	**N**	**%**	**N**	**%**	**N**	**%**	**N**	**%**	**N**	**%**
-2 to 0	96	*0.05*	-	-	102	*0.05*	89	*0.05*	52	*0.03*	275	*0.14*
0 to 2	320	*0.09*	-	-	258	*0.07*	222	*0.06*	141	*0.04*	736	*0.21*
2 to 4	297	*0.10*	-	-	219	*0.07*	240	*0.08*	107	*0.04*	652	*0.22*
4 to 6	282	*0.11*	-	-	228	*0.09*	295	*0.12*	113	*0.05*	676	*0.27*
6 to 8	245	*0.12*	-	-	194	*0.09*	277	*0.13*	107	*0.05*	603	*0.29*
8 to 10	200	*0.12*	-	-	162	*0.10*	243	*0.15*	110	*0.07*	535	*0.32*
10 to 12	152	*0.12*	-	-	137	*0.11*	202	*0.16*	83	*0.07*	418	*0.33*
12 to 14	95	*0.10*	-	-	116	*0.12*	153	*0.16*	58	*0.06*	324	*0.34*
14 to 16	71	*0.10*	-	-	103	*0.15*	117	*0.17*	47	*0.07*	245	*0.35*
**Year**												
2005-07	125	*0.08*	-	-	151	*0.10*	143	*0.08*	55	*0.03*	359	*0.23*
2006-08	140	*0.08*	-	-	115	*0.07*	148	*0.08*	54	*0.03*	332	*0.20*
2007-09	118	*0.07*	-	-	151	*0.09*	150	*0.08*	50	*0.03*	362	*0.21*
2008-10	159	*0.09*	-	-	157	*0.09*	182	*0.10*	80	*0.04*	415	*0.23*
2009-11	173	*0.09*	-	-	165	*0.09*	190	*0.09*	77	*0.04*	444	*0.24*
2010-12	202	*0.11*	-	-	152	*0.08*	187	*0.10*	94	*0.04*	480	*0.25*
2011-13	197	*0.11*	-	-	174	*0.10*	208	*0.11*	88	*0.05*	514	*0.28*
2012-14	203	*0.12*	-	-	146	*0.09*	180	*0.11*	107	*0.06*	486	*0.29*
2013-15	183	*0.13*	-	-	148	*0.10*	178	*0.12*	94	*0.06*	460	*0.32*
2014-16	133	*0.12*	-	-	76	*0.07*	134	*0.12*	61	*0.06*	313	*0.29*
2015-17	125	*0.14*	-	-	84	*0.09*	138	*0.15*	58	*0.07*	299	*0.34*

Tx= treatment; Sx = symptoms; Dx = diagnosis; * No codes were identified under this criterion for this mental illness.

4. **Mood disorders**

	**Diagnosis**		**Service use**		**Current Tx & Sx**		**Current Tx & historical Dx**		**Current Sx & historical Dx**		**Any**	
**Age-range**	**N**	**%**	**N**	**%**	**N**	**%**	**N**	**%**	**N**	**%**	**N**	**%**
-2 to 0	9703	*4.9*	2,270	*1.2*	8,621	*4.4*	16,966	*8.6*	6,390	*3.3*	22,308	*11.4*
0 to 2	34,087	*9.9*	8,611	*2.5*	25,459	*7.4*	46,248	*13.4*	17,914	*5.2*	59,753	*17.3*
2 to 4	19,882	*6.7*	7,339	*2.5*	19,444	*6.6*	40,975	*13.9*	14,249	*4.9*	50,274	*17.1*
4 to 6	15,480	*6.2*	6,280	*2.5*	15,833	*6.3*	36,750	*14.7*	12,173	*4.9*	43,906	*17.6*
6 to 8	12,046	*5.8*	5,317	*2.6*	12,791	*6.2*	31,967	*15.4*	10,124	*4.9*	37,537	*18.1*
8 to 10	8.987	*5.4*	4,430	*2.7*	9,980	*6.0*	26,581	*16.0*	8,090	*4.9*	30,726	*18.5*
10 to 12	6,620	*5.2*	3,473	*2.7*	7,492	*5.9*	21,002	*16.5*	6,119	*4.8*	24,077	*18.9*
12 to 14	4,832	*5.1*	2,537	*2.7*	5,601	*5.9*	16,058	*16.9*	4,657	*4.9*	18,238	*19.2*
14 to 16	3,639	*5.2*	1,817	*2.6*	4,148	*5.9*	12,188	*17.3*	3,491	*5.0*	13,754	*19.6*
**Year**												
2005-07	13,310	*8.4*	965	*0.6*	9,532	*6.0*	20,861	*13.1*	7,358	*4.6*	25,717	*16.2*
2006-08	13,260	*7.9*	1,638	*1.1*	10,012	*6.0*	22,173	*13.2*	8,027	*4.8*	22903	*16.3*
2007-09	12,700	*7.2*	1,978	*1.1*	10,605	*6.0*	23,387	*13.3*	8,412	*4.8*	24232	*16.9*
2008-10	12,505	*6.9*	2,585	*1.4*	11,150	*6.1*	24,681	*13.4*	9,892	*4.9*	25245	*16.2*
2009-11	12,505	*6.8*	2,929	*1.6*	11,807	*6.4*	25,832	*14.0*	9,218	*4.9*	26661	*16.9*
2010-12	12,259	*6.5*	3,260	*1.7*	12,108	*6.4*	26,817	*14.2*	8,771	*4.9*	27662	*17.0*
2011-13	10,996	*6.1*	3,815	*2.1*	11,714	*6.5*	26,413	*14.6*	7,785	*4.8*	27230	*17.5*
2012-14	9.572	*5.7*	6,118	*3.6*	10,569	*6.3*	24,519	*14.6*	6,738	*4.6*	25572	*17.5*
2013-15	7,794	*5.4*	7,389	*5.1*	9,265	*6.4*	22,248	*15.3*	6,738	*4.6*	23178	*18.3*
2014-16	5,773	*5.3*	6,206	*5.7*	6,963	*6.4*	17,144	*15.8*	4,996	*4.6*	18863	*19.0*
2015-17	4,487	*5.0*	5,191	*5.8*	5,644	*6.4*	14,660	*16.5*	3,987	*4.5*	15013	*19.7*

Tx= treatment; Sx = symptoms; Dx = diagnosis * No codes were identified under this criterion for this mental illness.

5. Neurotic disorders

	Diagnosis		Service use		Current Tx & Sx		Current Tx & historical Dx		Current Sx & historical Dx		Any	
**Age-range**	**N**	**%**	**N**	**%**	**N**	**%**	**N**	**%**	**N**	**%**	**N**	**%**
-2 to 0	7,021	*3.6*	42	*0.02*	1,135	*0.6*	4,423	*2.3*	2,085	*1.1*	10,712	*5.5*
0 to 2	14,982	*4.3*	113	*0.03*	2,356	*0.7*	8,287	*2.4*	4,351	*1.3*	21,739	*6.3*
2 to 4	13,090	*4.5*	85	*0.03*	2,526	*0.9*	9,198	*3.1*	4,138	*1.4*	20,630	*7.0*
4 to 6	11,691	*4.7*	82	*0.03*	2,507	*1.0*	9,420	*3.8*	3,987	*1.6*	19,237	*7.7*
6 to 8	9,735	*4.7*	72	*0.03*	2,204	*1.1*	8,981	*4.3*	3,415	*1.7*	17,019	*8.2*
8 to 10	7,932	*4.8*	69	*0.04*	1,791	*1.1*	8,078	*4.9*	2,847	*1.7*	14,395	*8.7*
10 to 12	6,158	*4.8*	32	*0.03*	1,427	*1.1*	6,827	*5.4*	2,232	*1.7*	11,599	*9.1*
12 to 14	4,654	*4.9*	24	*0.03*	1,072	*1.1*	5,578	*5.9*	1,706	*1.8*	9,088	*9.6*
14 to 16	3,415	*4.9*	12	*0.02*	827	*1.2*	4,364	*6.2*	1,352	*1.8*	6,962	*9.9*
**Year**												
2005-07	7,345	*4.6*	25	*0.02*	1,088	*0.7*	5,076	*3.2*	1,844	*1.2*	10,860	*6.8*
2006-08	7,130	*4.2*	37	*0.03*	1,254	*0.8*	5,463	*3.4*	2,067	*1.2*	11,307	*6.7*
2007-09	7,540	*4.3*	47	*0.03*	1,396	*0.8*	6,026	*3.4*	2,200	*1.3*	12,199	*6.9*
2008-10	7,643	*4.2*	60	*0.04*	1,535	*0.8*	6,324	*3.4*	2,374	*1.3*	12,724	*7.2*
2009-11	7,933	*4.3*	54	*0.04*	1,595	*0.9*	6,639	*3.6*	2,491	*1.4*	13,252	*7.3*
2010-12	8,145	*4.3*	54	*0.04*	1,678	*0.9*	6,884	*3.6*	2,788	*1.5*	13,732	*7.7*
2011-13	8,134	*4.5*	53	*0.04*	1,694	*0.9*	6,928	*3.8*	2,864	*1.6*	13,942	*7.7*
2012-14	7,574	*4.5*	46	*0.03*	1,698	*1.0*	6,653	*4.0*	2,843	*1.7*	13,162	*7.8*
2013-15	7,024	*4.8*	26	*0.02*	1,481	*1.0*	6,073	*4.2*	2,555	*1.8*	12,151	*8.4*
2014-16	5,389	*5.0*	29	*0.03*	1,293	*1.2*	4,212	*4.5*	2,137	*2.0*	9,605	*8.9*
2015-17	4,789	5.3	9	*0.01*	1,133	*1.3*	3662	*4.7*	1688	*2.3*	8,447	*9.5*

Tx= treatment; Sx = symptoms; Dx = diagnosis;

6. Eating disorders

	Diagnosis		Service use		Current Tx & Sx*		Current Tx & historical Dx*		Current Sx & historical Dx		Any	
**Age-range**	**N**	**%**	**N**	**%**	**N**	**%**	**N**	**%**	**N**	**%**	**N**	**%**
-2 to 0	257	*0.12*	48	*0.02*	-	-	-	-	10	*0.01*	299	*0.15*
0 to 2	405	*0.12*	72	*0.02*	-	-	-	-	10	*0.00*	455	*0.13*
2 to 4	382	*0.13*	62	*0.02*	-	-	-	-	14	*0.00*	430	*0.14*
4 to 6	307	*0.13*	53	*0.02*	-	-	-	-	23	*0.01*	354	*0.12*
6 to 8	224	*0.11*	44	*0.02*	-	-	-	-	9	*0.00*	254	*0.12*
8 to 10	175	*0.11*	37	*0.02*	-	-	-	-	6	*0.00*	194	*0.10*
10 to 12	115	*0.10*	19	*0.01*	-	-	-	-	4	*0.00*	130	*0.09*
12 to 14	75	*0.09*	11	*0.01*	-	-	-	-	4	*0.00*	83	*0.09*
14 to 16	54	*0.08*	6	*0.01*	-	-	-	-	3	*0.00*	62	*0.09*
**Year**												
2005-07	204	*0.13*	21	*0.02*	-	-	-	-	3	*0.00*	224	*0.14*
2006-08	202	*0.12*	38	*0.02*	-	-	-	-	4	*0.00*	226	*0.13*
2007-09	271	*0.15*	41	*0.02*	-	-	-	-	6	*0.00*	303	*0.17*
2008-10	246	*0.13*	40	*0.02*	-	-	-	-	4	*0.00*	270	*0.15*
2009-11	224	*0.12*	39	*0.02*	-	-	-	-	16	*0.01*	259	*0.14*
2010-12	217	*0.11*	40	*0.02*	-	-	-	-	7	*0.00*	241	*0.13*
2011-13	169	*0.09*	30	*0.02*	-	-	-	-	9	*0.00*	192	*0.11*
2012-14	159	*0.09*	39	*0.02*	-	-	-	-	7	*0.00*	192	*0.11*
2013-15	128	*0.09*	33	*0.02*	-	-	-	-	7	*0.00*	152	*0.10*
2014-16	99	*0.09*	19	*0.01*	-	-	-	-	12	*0.01*	113	*0.10*
2015-17	75	*0.08*	11	*0.02*	-	-	-	-	8	*0.01*	89	*0.10*

Tx= treatment; Sx = symptoms; Dx = diagnosis; * No codes were identified under this criterion for this mental illness.

7. Personality disorders

	Diagnosis		Service use*		Current Tx & Sx*		Current Tx & historical Dx*		Current Sx & historical Dx*		Any	
**Age-range**	**N**	**%**	**N**	**%**	**N**	**%**	**N**	**%**	**N**	**%**	**N**	**%**
-2 to 0	158	*0.08*	-	-	-	-	-	-	-	-	332	*0.08*
0 to 2	301	*0.09*	-	-	-	-	-	-	-	-	402	*0.09*
2 to 4	260	*0.09*	-	-	-	-	-	-	-	-	286	*0.09*
4 to 6	230	*0.09*	-	-	-	-	-	-	-	-	249	*0.09*
6 to 8	178	*0.08*	-	-	-	-	-	-	-	-	174	*0.09*
8 to 10	132	*0.07*	-	-	-	-	-	-	-	-	121	*0.08*
10 to 12	87	*0.07*	-	-	-	-	-	-	-	-	75	*0.07*
12 to 14	67	*0.07*	-	-	-	-	-	-	-	-	55	*0.07*
14 to 16	36	*0.05*	-	-	-	-	-	-	-	-	22	*0.05*
**Year**												
2005-07	96	*0.06*	-	-	-	-	-	-	-	-	77	*0.06*
2006-08	79	*0.05*	-	-	-	-	-	-	-	-	67	*0.05*
2007-09	92	*0.05*	-	-	-	-	-	-	-	-	79	*0.05*
2008-10	129	*0.07*	-	-	-	-	-	-	-	-	107	*0.07*
2009-11	127	*0.07*	-	-	-	-	-	-	-	-	104	*0.07*
2010-12	153	*0.08*	-	-	-	-	-	-	-	-	137	*0.08*
2011-13	150	*0.08*	-	-	-	-	-	-	-	-	131	*0.08*
2012-14	149	*0.09*	-	-	-	-	-	-	-	-	147	*0.09*
2013-15	168	*0.12*	-	-	-	-	-	-	-	-	156	*0.12*
2014-16	156	0.14	-	-	-	-	-	-	-	-	145	0.14
2015-17	150	0.17	-	-	-	-	-	-	-	-	126	0.17

Tx= treatment; Sx = symptoms; Dx = diagnosis; * No codes were identified under this criterion for this mental illness.

8. Alcohol misuse

	Diagnosis		Service use		Current Tx & Sx		Current Tx & historical Dx		Current Sx & historical Dx		Any	
**Age-range**	**N**	**%**	**N**	**%**	**N**	**%**	**N**	**%**	**N**	**%**	**N**	**%**
-2 to 0	191	*0.10*	81	*0.04*	1	*0.00*	46	*0.02*	5	*0.00*	263	*0.13*
0 to 2	295	*0.09*	141	*0.04*	4	*0.00*	74	*0.02*	15	*0.00*	414	*0.12*
2 to 4	377	*0.13*	183	*0.06*	3	*0.00*	95	*0.03*	12	*0.00*	517	*0.18*
4 to 6	398	*0.16*	177	*0.07*	3	*0.00*	107	*0.04*	13	*0.01*	551	*0.22*
6 to 8	415	*0.20*	210	*0.10*	2	*0.00*	137	*0.07*	11	*0.01*	585	*0.28*
8 to 10	389	*0.23*	181	*0.11*	2	*0.00*	130	*0.08*	17	*0.01*	550	*0.33*
10 to 12	348	*0.27*	178	*0.14*	1	*0.00*	123	*0.08*	18	*0.01*	480	*0.38*
12 to 14	287	*0.30*	144	*0.15*	4	*0.00*	105	*0.10*	15	*0.02*	382	*0.40*
14 to 16	233	*0.33*	98	*0.14*	2	*0.00*	78	*0.11*	15	*0.02*	316	*0.45*
**Year**												
2005-07	226	*0.14*	107	*0.09*	4	*0.00*	71	*0.04*	9	*0.01*	308	*0.19*
2006-08	274	*0.16*	128	*0.08*	1	*0.00*	83	*0.05*	9	*0.01*	363	*0.21*
2007-09	311	*0.18*	129	*0.08*	1	*0.00*	78	*0.05*	9	*0.01*	411	*0.24*
2008-10	333	*0.18*	165	*0.08*	1	*0.00*	94	*0.05*	15	*0.01*	472	*0.26*
2009-11	282	*0.15*	160	*0.08*	1	*0.00*	85	*0.05*	7	*0.00*	403	*0.22*
2010-12	349	*0.18*	150	*0.08*	4	*0.00*	97	*0.05*	24	*0.01*	475	*0.25*
2011-13	299	*0.16*	131	*0.08*	2	*0.00*	91	*0.05*	14	*0.01*	413	*0.23*
2012-14	278	*0.16*	153	*0.09*	6	*0.00*	95	*0.06*	10	*0.01*	398	*0.23*
2013-15	228	*0.16*	124	*0.09*	3	*0.00*	76	*0.05*	7	*0.01*	328	*0.24*
2014-16	202	*0.19*	73	*0.08*	0	*0.00*	73	*0.07*	5	*0.01*	268	*0.23*
2015-17	151	*0.17*	73	*0.08*	0	*0.00*	52	*0.06*	5	*0.01*	219	*0.25*

Tx= treatment; Sx = symptoms; Dx = diagnosis; * No codes were identified under this criterion for this mental illness.

9. Substance misuse

	Diagnosis		Service use		Current Tx & Sx		Current Tx & historical Dx		Current Sx & historical Dx		Any	
**Age-range**	**N**	**%**	**N**	**%**	**N**	**%**	**N**	**%**	**N**	**%**	**N**	**%**
-2 to 0	379	*0.19*	161	*0.08*	10	*0.01*	121	*0.06*	27	*0.01*	505	*0.26*
0 to 2	561	*0.16*	274	*0.08*	11	*0.00*	202	*0.06*	32	*0.01*	770	*0.22*
2 to 4	540	*0.18*	268	*0.10*	11	*0.00*	195	*0.07*	35	*0.01*	742	*0.25*
4 to 6	461	*0.18*	240	*0.10*	9	*0.00*	164	*0.07*	17	*0.01*	639	*0.26*
6 to 8	363	*0.17*	169	*0.09*	9	*0.00*	122	*0.06*	22	*0.01*	491	*0.24*
8 to 10	289	*0.17*	141	*0.10*	3	*0.00*	87	*0.05*	15	*0.01*	403	*0.24*
10 to 12	184	*0.14*	97	*0.09*	4	*0.00*	73	*0.06*	8	*0.01*	261	*0.21*
12 to 14	138	*0.15*	66	*0.09*	3	*0.00*	45	*0.05*	9	*0.01*	191	*0.20*
14 to 16	82	*0.12*	33	*0.06*	0	*0.00*	24	*0.03*	0	*0.00*	105	*0.15*
**Year**												
2005-07	229	*0.17*	144	*0.09*	7	*0.00*	106	*0.07*	26	*0.02*	372	*0.23*
2006-08	240	*0.17*	131	*0.08*	4	*0.00*	93	*0.06*	20	*0.01*	388	*0.23*
2007-09	295	*0.19*	146	*0.08*	6	*0.00*	108	*0.06*	22	*0.01*	438	*0.25*
2008-10	261	*0.17*	138	*0.08*	6	*0.00*	120	*0.07*	14	*0.01*	452	*0.22*
2009-11	278	*0.17*	147	*0.08*	7	*0.01*	121	*0.06*	15	*0.01*	419	*0.23*
2010-12	254	*0.15*	156	*0.08*	7	*0.00*	120	*0.06*	17	*0.01*	358	*0.23*
2011-13	262	*0.17*	150	*0.08*	10	*0.01*	117	*0.06*	17	*0.01*	223	*0.25*
2012-14	275	*0.18*	156	*0.09*	7	*0.00*	103	*0.06*	14	*0.01*	184	*0.25*
2013-15	246	*0.19*	129	*0.09*	6	*0.00*	73	*0.05*	1	*0.00*	322	*0.25*
2014-16	153	*0.16*	82	*0.08*	0	*0.00*	42	*0.04*	5	*0.01*	223	*0.21*
2015-17	109	*0.14*	70	*0.08*	0	*0.00*	30	*0.03*	4	*0.01*	184	*0.21*

Tx= treatment; Sx = symptoms; Dx = diagnosis;

Appendix 2: Data quality

HH compiled lists of diagnoses, mental health services and therapies using existing lists of codes and additional bespoke search strategies, these resources and search strategies are detailed in Appendix A. To ensure we obtained all relevant records, we reviewed the medical and therapy histories of mother with definitive mental illness and added any new relevant codes (see clinicalcodes.org for full list of codes). KA and ES are trained psychiatrists and were consulted on the inclusion of specific Read Codes. In addition, ES identified the symptoms core to the clinical presentation of specific mental illness(es) and these were rigorously reviewed by KA. JN and RK are pharmacists familiar with general practice dispensing and were consulted on inclusion of specific product names. Appendix 3 details the source of data and type of code available to identify mental disorders.

Appendix 3: Resources and searches used to identify Read codes and Prod codes for case definition

Table 1 Resources and searches used to identify Read codes for case definition

Mental Illness	Source
All disorders	Mental Health Dictionary created by co-author Matt Carr
Alcohol dependence	Alcohol abuse codes developed from the Scottish ISD grouping by Dr Duncan Edwards with Dr Lucy Gerza of the Cambridge CPRD group
Substance dependence	Psychoactive substance misuse (NOT ALCOHOL) List created by CPRD@Cambridge
Mood disorders	Depression List created by CPRD@Cambridge Dr Gerza, Anna Cassell, Dr Edwards<br>Depression readcodes from Lu et al. 2012 (PhD project)<br>363 - res36: Depression readcodes from clincalcodes.org<br>Depression and anxiety codes from John et al. 2016<br>HH generated search stubs from sources above: (readcodes <7chars) i.e. E2003 1B17. 1B1U. 1BQ.. 1BT.. 1BT.. 1BU.. E112 E113 E135 E2B EU32 EU33 EU34 EU41
Neurotic	Anxiety codes developed from the Scottish ISD list by Dr Duncan Edwards.<br>Anxiety list of codes from Clinicalcodes.org Windfuhr et al. Suicide risk 2016.<br>Depression and anxiety codes from John et al. 2016<br>365 - res36: Anxiety readcodes from clincalcodes.org<br>Neurotic stubs: HH Generated from above sources (readcodes <7chars) 1B12 1B13 2258 2259 E200 EU41 R2y2

Serious mental illness	Bipolar readcodes from Lu et al. 2012 (PhD project)<br>362 - res36: Bipolar disorder readcodes from clincalcodes.org<br>Bipolar stubs:HH Generated from above sources i.e. 6657 BY..00<br>Schizophrenia readcodes from Lu et al. 2012 (PhD project)<br>Serious mental illness res26 readcodes from clincalcodes.org<br>Reilly et al. searched for serious mental illness including mental health act codes utilising:<br>Stubs: E1 E2122 Eu2 Eu3 212t 212X 1BH 1464 665 146 9H<br>Terms: chiz paranoi ebephreni elusion sychos sychot mania Mania Manic manic ipolar i-polar
Eating disorders	364 - res36: Eating disorder readcodes from clincalcodes.org<br>Generated from D13 (readcodes <7chars) 1612 1614 R036
Personality disorders	Code list: res38: Personality disorder from Olier et al. 2015<br>Perez et al. Identifies serious mental illness codes including personality disorders E10.. Eu31. E2B.. Eu33. E200. E202. EU41 Eu40 E21.. Eu340 Eu341 Eu21. Eu6..
Psychiatric disorder NOS	HH search strategy based on general mental health terms: psych Psych mental Mental therap Therap

Table 2 Resources and searches used to identify Product codes for case definition

Table	Origin
All psychotherapies	Drug Dictionary created by co-author Matt CarrDrug dictionary created by Cathy Morgan https://www.bmj.com/content/359/bmj.j4351<br>HH search strategy based on BNF chapters and drug substances

Mood stabilisers	Lithium drug list developed by Anna Cassell and Duncan Edwards
Antidepressants	Anti-depressant list developed by Cambridge – Dr Gerza, Anna Cassell, Dr Edwards
Anxiolytics	List was created using chapter 4.1.1 (Hypnotics and Anxiolytics) in the BNF, but it excluded melatonin, promethazine, oxybate, chloral hydrate, chlorodiazepoxide. It also included the anxiolytics in chapter 4.1.2 and trazadone. Does not include SSRIs or tricyclics.
Serious mental illness	Drugs for the treatment of severe mental illness. Hardoon S, Hayes JF, Blackburn R, Petersen I, Walters K, Nazareth I, et al. (2013) Recording of Severe Mental Illness in United Kingdom Primary Care, 2000–2010. PLoS ONE8(12): e82365. https://doi.org/10.1371/journal.pone.0082365

Appendix 4: Methods and models used for inverse probability of missingness weights

Using the language of Rubin(Rubin, 1976), we considered losses to follow-up due to the study period ending or the practice ceasing to collect data as Missing Completely At Random (MCAR) and we considered loss due to the mother transferring out of a practice might be Missing At Random (MAR). Therefore, we sought to adjust for this by calculating inverse probability weighting using methodology adapted from Howe et al(Howe, Cole, Lau, Napravnik, & Eron, 2016).

Each child's follow-up was split into 2 year periods (0-2 years, 2-4 years etc.) using all available follow-up (rather than censoring at the first outcome event), and an indicator variable was created if the child was censored in that period because their mother transferred out of the clinical practice. The probability that the child was censored due to this was calculated using logistic regression models with variables identified in the mother's primary care record.

Static variables used to create weights were:

- Maternal age at birth (continuous and its square)
- Practice-level IMD quintile (categorical variable)
- Geographical region of the UK
- Days enrolled at practice prior to birth (continuous and its square)

Dynamic variables used were:

- Age of child during start of period (continuous and its square)
- Mental illness in the two years prior to the period, using categories as specified in the paper

Results from the logistic regression model used to predict completeness over follow-up is shown in the below table

Variable	Odds Ratio	95% CI
Age of child at beginning of period (years):		
Continuous	0.875	0.874, 0.876
Square	0.987	0.987, 0.987
Age of mother at birth:		
Continuous	1.007	1.006, 1.008
Square	0.999	0.999, 0.999
Practice-level IMD quintile:		
Highest	Ref	
2nd	0.881	0.871, 0.891
3rd	0.867	0.857, 0.877
4th	0.810	0.801, 0.819
Lowest	0.831	0.821, 0.840
Geographical region of UK:		
North East	0.841	0.819, 0.864
North West	Ref	
Yorkshire & The Humber	0.618	0.604, 0.633
East Midlands	0.450	0.440, 0.461
West Midlands	0.938	0.924, 0.953
East of England	0.781	0.769, 0.794
South West	0.914	0.900, 0.928
London	0.755	0.743, 0.766
South East Coast	0.956	0.944, 0.968
Northern Ireland	1.256	1.232, 1.282
Scotland	1.060	1.044, 1.075
Wales	1.202	1.184, 1.220
Time enrolled prior to birth (days):		
Continuous	1.023	1.023, 1.024
Square	0.999	0.999, 0.999
Mental disorder in the prior period:		
Non-affective psychosis	0.888	0.812, 0.972
Affective psychosis	0.915	0.855, 0.979
Mood disorder	0.891	0.882, 0.900
Neurotic disorder	0.925	0.912, 0.938
Eating disorder	1.116	1.013, 1.230
Behavioural disorder	0.653	0.583, 0.730
Alcohol misuse	1.018	0.947, 1.093
Substance misuse	0.926	0.863, 0.993
MD not otherwise specified	0.943	0.914, 0.974

* Missing data were due to insufficient data in the 2 years prior to birth
Continuous variables were centred before being entered into the model.

The resulting individual predicated probabilities were used to calculate cumulative and stabilised weights. The period prevalence of any maternal mental disorder using four different analysis (unadjusted, adjusted for year, and adjusted for weights and stabilised weights) are shown in the table below.

	**Unadjusted**		**Unweighted**		**Weighted**		**Stabilised weighted**	
**Age-group**	**Statistic**	**95% CI**	**Statistic**	**95% CI**	**Statistic**	**95% CI**	**Statistic**	**95% CI**
0 to 2	21.6	21.4-21.7	21.7	21.5-21.8	22.4	22.2-22.6	21.9	21.7-22.1
2 to 4	21.7	21.6-21.9	21.8	21.6-22.0	22.6	22.4-22.8	22.2	22.0-22.3
4 to 6	22.3	22.2-22.5	22.4	22.2-22.6	23.2	23.0-23.5	22.8	22.7-23.0
6 to 8	23.0	22.7-23.2	22.9	22.7-23.1	24.0	23.7-24.2	23.6	23.4-23.8
8 to 10	23.4	23.1-23.6	23.3	23.0-23.5	24.5	24.3-24.8	24.2	23.9-24.4
10 to 12	23.9	23.6-24.1	23.7	23.4-24.0	25.3	25.0-25.6	25.0	24.7-25.2
12 to 14	24.2	23.9-24.5	24.0	23.7-24.3	26.4	26.0-26.8	25.9	25.6-26.3
14 to 16	24.6	24.2-24.9	24.4	24.1-24.8	27.8	27.3-28.3	27.3	26.8-27.8
